# A Case of Primary Gastric Carcinosarcoma with Small Intestine Metastasis

**DOI:** 10.70352/scrj.cr.25-0303

**Published:** 2025-10-16

**Authors:** Chikara Mashiba, Shinichi Kinami, Yuta Sannomiya, Shota Motoyama, Hitoshi Saito, Sohsuke Yamada

**Affiliations:** Department of General and Gastroenterologic Surgery, Kanazawa Medical University Himi Municipal Hospital, Himi, Toyama, Japan

**Keywords:** primary gastric carcinosarcoma, local resection, small intestinal intraluminal metastasis, capecitabine, irinotecan

## Abstract

**INTRODUCTION:**

Primary gastric carcinosarcoma is extremely rare. Herein, we report a case of primary gastric carcinosarcoma with small intestinal intraluminal metastasis.

**CASE PRESENTATION:**

The patient was a 77-year-old man who was referred to our hospital for further examination of occult fecal blood. At this time, the patient reported lightheadedness, and severe anemia was confirmed, with a hemoglobin level of 6.5 g/dL. Upper gastrointestinal endoscopy revealed a mass with bleeding spots in the middle 3rd of the stomach, which was thought to be the cause of the anemia. The patient’s overall condition was poor; therefore, we decided to limit the surgery to local resection of the primary lesion as palliative treatment, with the main goal of controlling bleeding. The final pathological diagnosis was gastric carcinosarcoma. Postoperatively, the progression of anemia stopped, the patient was able to eat without any problems, and he was discharged home. However, 9 days later, the patient visited the emergency department complaining of abdominal pain. He was diagnosed with intestinal obstruction and underwent surgery. During surgery, a hard mass was palpable in the small intestine, and the lesion was resected. The pathological findings of the small intestinal mass were identical to those of the gastric tumor, and the patient was diagnosed with small intestinal intraluminal metastasis of the gastric carcinosarcoma. Eight months after surgery, pulmonary metastasis was detected by a CT scan. Chemotherapy with capecitabine and irinotecan was initiated, and tumor reduction was achieved.

**CONCLUSIONS:**

Primary gastric carcinosarcoma may present with intraluminal metastasis of the small intestine, and clinicians should make a note of this when treating such patients.

## Abbreviations

5-FU
: 5-fluorouracil
CDDP
: cisplatin
CPT-11
: irinotecan
FEV
: forced expiratory volume
VC
: vital capacity

## INTRODUCTION

Carcinosarcoma is a tumor that contains both epithelial malignancy and non-epithelial malignant sarcomas.^1)^ Primary gastric carcinosarcoma is rare; since Queckenstedt’s first report,^2)^ fewer than 100 cases have been reported to date.^3–8)^

Small intestinal metastases of gastric tumors are also rare.^9,10)^ Possible routes of metastasis include peritoneal, hematogenous, lymphatic, and intraluminal metastasis.^11)^ However, intraluminal metastases are extremely rare.^9)^

We encountered a case of primary gastric carcinosarcoma complicated by intraluminal metastasis to the small intestine. This is an extremely rare case, which we report here.

## CASE PRESENTATION

**Fig. 1:**
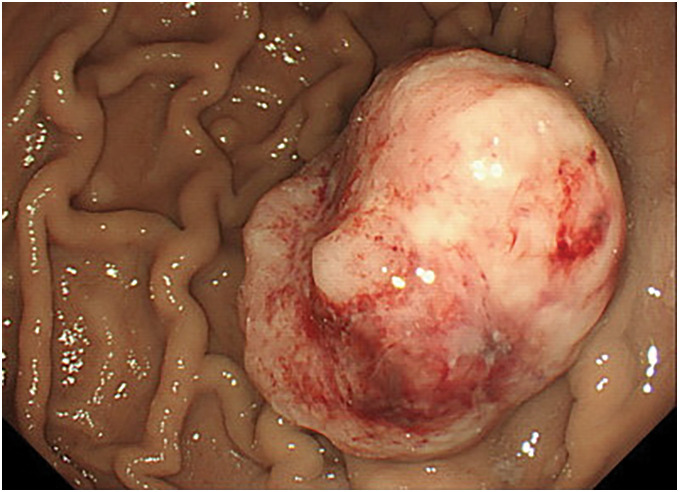
An upper gastrointestinal endoscopy revealed a mass with bleeding marks on the posterior wall of the greater curvature of the middle portion of the stomach.

**Fig. 2:**
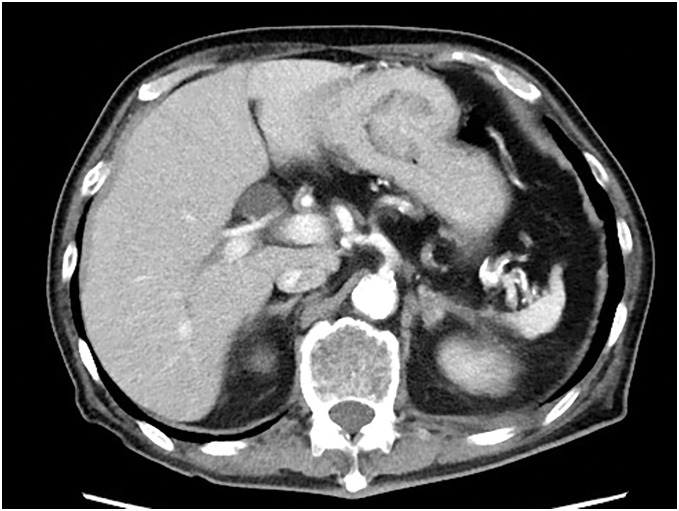
An abdominal CT scan showed a tumor with no serosal invasion, no lymph node enlargement, and no distant metastasis.

**Fig. 3:**
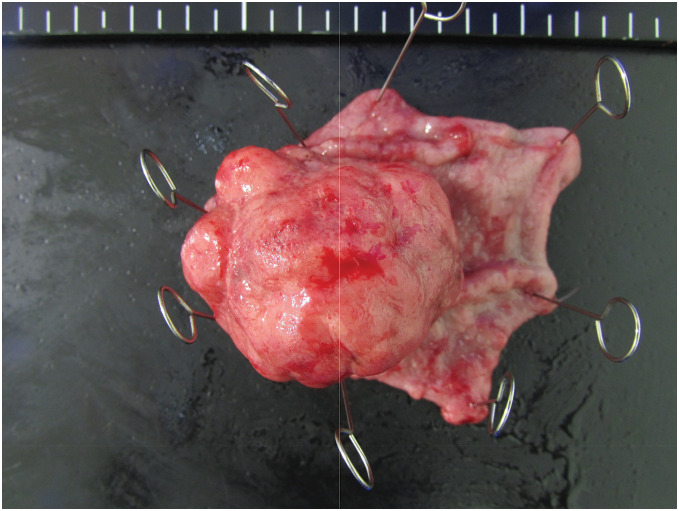
The picture of the resected specimen. It is a 4.0-cm tumor lesion.

**Fig. 4:**
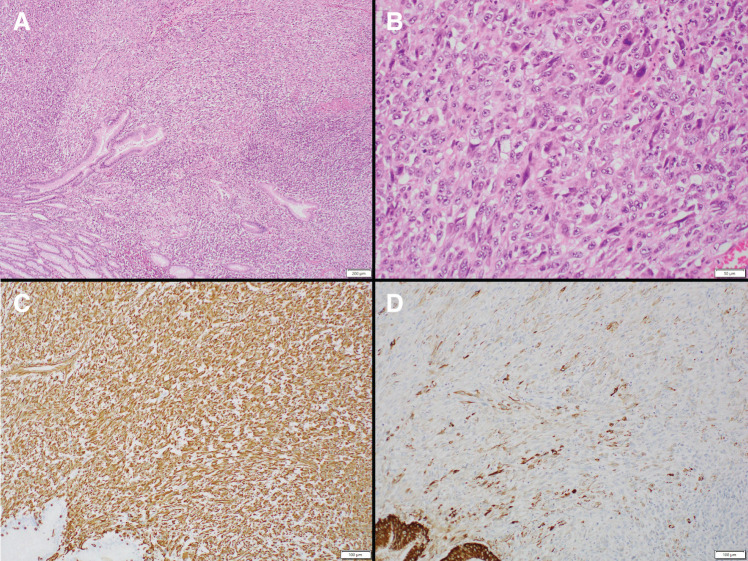
Pathological findings revealed round to spindle-shaped tumor lesions with a high N/C ratio. These lesions were arranged in a sarcoma-like growth pattern and extended into the deep submucosa of the small intestine with necrosis. (**A**, **B**) Immunohistochemical staining showed that the tumor cells were strongly positive for vimentin and partially positive for CAM 5.2 (**C**, **D**). N/C ratio, nuclear-to-cytoplasmic ratio

The patient was a 77-year-old man who was referred to our hospital for further examination of occult fecal blood. His medical history included chronic obstructive pulmonary disease, hypertension, atrial fibrillation, angina pectoris, and arteriosclerosis obliterans of the left lower limb. First, a total colonoscopy was performed on a day in October 2013, during which two adenomas measuring 4 mm were endoscopically resected. However, at this time, the patient reported lightheadedness, and severe anemia was confirmed with a hemoglobin level of 6.5 g/dL; therefore, he was admitted to the internal medicine department of our hospital. Upper gastrointestinal endoscopy revealed a mass with bleeding spots on the posterior wall near the greater curvature of the middle third of the stomach, which was thought to be the cause of the anemia (**Fig. 1**). Biopsy revealed highly atypical spindled and epithelioid cells with enlarged and irregular hyperchromatic nuclei, admixed with a few bizarre cells and abnormal mitotic figures, arranged predominantly in a sarcomatoid pattern. The tumor was diagnosed as primary carcinosarcoma. Abdominal CT revealed a tumor in the same area of the stomach; however, no serosal invasion, lymph node enlargement, or distant metastasis was observed (**Fig. 2**). Blood samples revealed malnourishment (**Table 1**). Respiratory function tests revealed restrictive ventilatory disorder with a %VC of 59.5% and FEV1.0 of 84.8%. Based on these results, the patient’s overall condition was determined to be poor. Usually, extensive gastrectomy with lymph node dissection, which is the standard surgical procedure, is performed. However, because this strategy would be highly invasive and there was doubt about the patient’s ability to tolerate the procedure, we decided to limit the surgery to local resection of the primary lesion as a palliative treatment, with the main goal of controlling bleeding, while taking into consideration the patient’s general condition. Local gastric resection was performed 48 days after admission (**Fig. 3**). Pathological findings revealed a round to spindle-shaped tumor lesion with a high N/C ratio. These lesions were arranged in a sarcomatous growth pattern, extended into the deep submucosa of the stomach, and were accompanied by necrosis. Immunohistochemical staining revealed that the tumor cells were strongly positive for vimentin and partially positive for CAM 5.2, confirming the final diagnosis of gastric carcinosarcoma (**Fig. 4**). After surgery, the progression of anemia stopped, the patient was able to eat without any problems, and he was discharged home 15 days after surgery.

**Table 1:** Blood analysis

WBC	31.0 × 10^3^/μL	AST	17 IU/L
RBC	1.73 × 10^6^/μL	ALT	21 IU/L
Hb	5.2 g/dL	γ-GTP	27 IU/L
Ht	17.2%	Alb	2.1 g/dL
PLT	338 × 10^3^/μL	TP	4.6 g/dL
Fe	11 μg/dL	T-bil	0.25 mg/dL
CRP	10.62 mg/dL	D-bil	0.12 mg/dL
PT	87%	BUN	16.3 mg/dL
APTT	39.8 s	Cre	0.74 mg / dL
Fibrinogen	361 mg/dL	Na	138 mEq/L
D-dimer	0.59 μg/dL	K	3.4 mEq/L

γ-GTP, γ-glutamyl transpeptidase; Alb, albumin; ALT, alanine aminotransferase; APTT, activated partial thromboplastin time; AST, aspartate aminotransferase; BUN, blood urea nitrogen; Cre, creatinine; CRP, C-reactive protein; D-bil, direct bilirubin; Fe, iron; Hb, hemoglobin; Ht, hematocrit; K, potassium; Na, sodium; PLT, platelet; PT, prothrombin time; RBC, red blood cell; T-bil, total bilirubin; TP, total protein; WBC, white blood cell

**Fig. 5:**
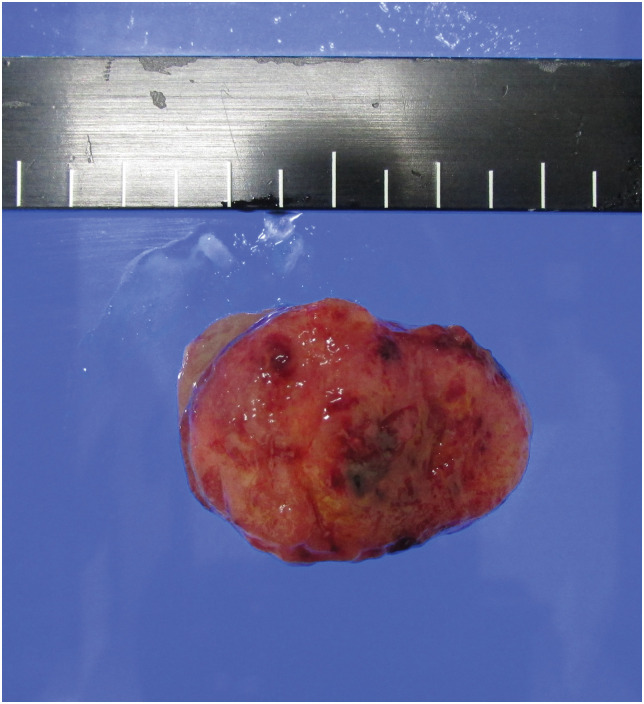
Resected specimen showing small intestinal intraluminal metastasis.

**Fig. 6:**
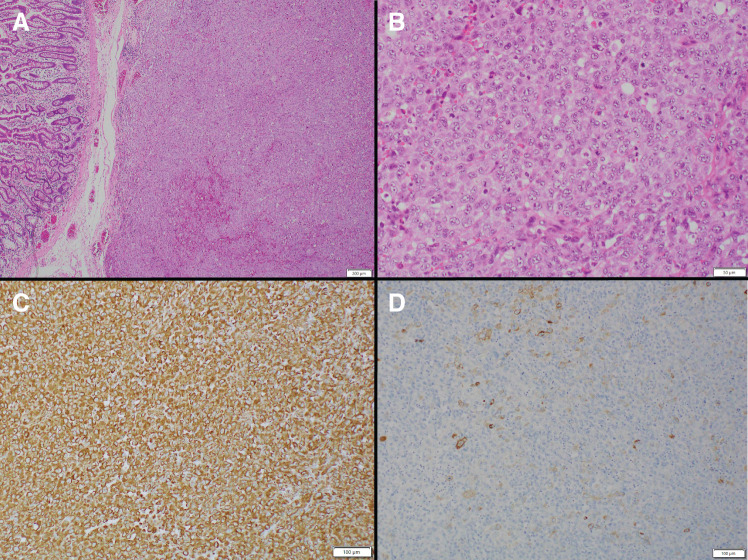
Pathological image of the small intestinal tumor. No difference was observed compared to the primary tumor. (**A**) H&E, low magnification. (**B**) H&E, high magnification. (**C**) Vimentin immunostaining. (**D**) CAM 5.2 immunostaining. H&E, hematoxylin and eosin

However, 9 days later, the patient visited the emergency department complaining of abdominal pain. The patient was found to have anemia and a niveau on abdominal radiography, and was admitted to the hospital urgently with a diagnosis of intestinal obstruction. Although an ileal tube was inserted and decompression was attempted, there was little improvement in the symptoms. Therefore, surgery was performed 6 days after admission. A strangulated bowel obstruction due to adhesions was observed, and ischemic areas of the small intestine and mesentery were resected. During surgery, a hard mass was palpable in the small intestine, 30 cm from the oral side of the resection site. During the first surgery, the laparotomy was too limited to prevent invasion, and a complete assessment of the abdominal cavity was not conducted. A larger laparotomy was performed during the second surgery, and the entire abdominal cavity was examined to confirm the absence of peritoneal dissemination. Additionally, no obstruction due to the tumor was observed. Therefore, local full-thickness resection of the intestinal tract where the tumor was located was performed. We did not perform an imaging diagnosis before the second surgery; therefore, we did not consider the possibility of a small intestinal tumor. In addition, imaging findings before the first surgery were unclear. The pathological findings of the small intestinal mass were identical to those of the gastric tumor, and the patient was diagnosed with small intestinal intraluminal metastasis of the gastric carcinosarcoma (**Fig. 5**). No pathological differences were observed compared to the primary tumor (**Fig. 6**). After surgery, oral intake gradually improved, and the patient was discharged 30 days later.

**Fig. 7:**
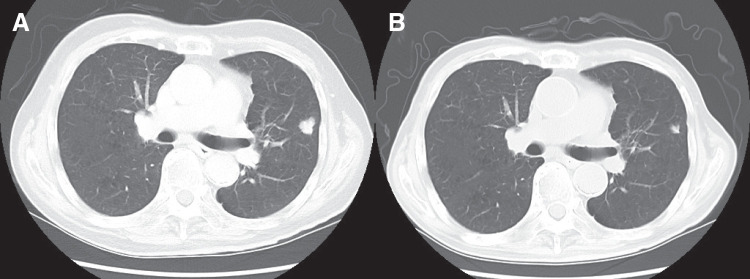
(**A**) CT scan on a day in August 2024, revealed lung metastases. (**B**) A reduction in the size of the metastatic lesions was observed after chemotherapy.

Initially, the patient was followed up without chemotherapy during outpatient visits, but lung metastasis was discovered on a CT scan on a day in August 2024. Unfortunately, PET-CT was not available. Chemotherapy with capecitabine and irinotecan was initiated 17 days after diagnosis of the recurrence. CPT-11 (irinotecan) was administered at 150 mg/m^2^ on day 1, and capecitabine was administered at 2400 mg/body every 2 for 3 weeks (**Fig. 7A**). Follow-up CT showed a reduction in the metastatic lesions, and the patient was diagnosed with RECIST PR. As of May 2025, there has been no progression, and chemotherapy is ongoing (**Fig. 7B**).

## DISCUSSION

Herein, we report the case of a patient with primary gastric carcinosarcoma. A unique feature of this case was the development of intraluminal metastasis to the small intestine. To the best of our knowledge, there are no previous reports of intraluminal metastasis in primary gastric carcinosarcoma. This case is also interesting in that, although the patient developed a recurrence of pulmonary metastasis, chemotherapy using capecitabine and irinotecan was effective.

Carcinosarcoma is a mixture of epithelial malignant tumors, adenocarcinomas, and non-epithelial malignant tumors, sarcomas, within the same tumor.^1)^ It is relatively common in organs such as the uterus, ovaries, bladder, lungs, and esophagus.^8,11)^ On the other hand, primary gastric carcinosarcoma is very rare.^3–8)^

There are 4 hypotheses regarding the etiology of primary gastric carcinosarcoma^12)^: 1) collision tumor theory, 2) composition tumor theory, 3) combination tumor theory, and 4) metaplastic tumor theory. The collision tumor theory is a hypothesis that carcinosarcomas are formed when a collision tumor occurs due to the simultaneous malignant transformation of epithelial and stromal tissues. The composition tumor theory is a hypothesis that carcinosarcomas are formed due to a pseudosarcoma-like reaction of the stroma to cancer. The combination tumor theory is a hypothesis that carcinosarcomas are formed when a single clone contains both carcinoma and sarcoma components because of the stem cell-like multi-differentiation ability of tumor cells. According to the metaplastic tumor theory, sarcomatous elements undergo metaplasia secondary to cancer. Currently, tumors that fit the collision tumor theory are excluded from the narrow definition of carcinosarcomas. The most likely theory is the combination tumor theory, which states that most carcinosarcomas originate from a single cell based on clonality analysis and differentiate into components that exhibit epithelial elements and components that exhibit sarcomatous elements during the tumor development process.^13)^

Primary gastric carcinosarcoma is highly malignant and has a poor prognosis due to early hematogenous metastasis and recurrence. The average survival time has been reported to be about 7–10 months.^3,8,14–16)^ Early surgical complete resection may be necessary for long-term survival. Radical gastrectomy, consisting of extensive gastrectomy and lymph node dissection, is considered the only treatment option for gastric carcinosarcoma.^17)^ However, even after radical gastrectomy, more than 50% of cases recur within one year after surgery, with the majority of recurrences occurring in the peritoneum.^14,18–20)^ The frequency of lymph node and liver metastases is unknown, and to the best of our knowledge, there has not been a single reported case of small intestinal metastasis. In this case, the patient had anemia and malnutrition due to bleeding and protein leakage from the tumor, and the patient’s general condition was very poor before surgery. Therefore, it was predicted that the patient would not be able to tolerate standard surgery. As a result, local resection was chosen as a palliative surgical option. Although the patient ultimately experienced recurrence due to lung metastasis, there was no lymph node recurrence. Depending on the patient’s general condition, a treatment plan that limits the patient to only primary tumor resection by local resection may be an option.

In carcinosarcoma metastasis, both carcinoma and sarcoma components may be found in the metastatic lesion; however, either components may also be found separately. The ratio of carcinoma to sarcoma components and the degree of tissue differentiation vary from case to case. It is presumed that the potential for lymph node metastasis also differs. The reasons why the components vary depending on the metastatic organ include the difference in the time at which the carcinoma and sarcoma components become malignant, the fact that their vascular invasion formation is independent, and that one of the components is more suitable for development and growth at the metastatic site.^21)^ In gastric carcinosarcomas, both adenocarcinoma and sarcoma components have the potential to metastasize.^22)^ There have been cases in which the adenocarcinoma metastasized to the lymph nodes,^23)^ cases in which only adenocarcinoma components were found in metastatic lymph nodes and carcinosarcoma components were found in liver metastases,^24)^ and cases in which only sarcoma cells were found in aortic intimal metastasis.^25)^

On the other hand, metastasis from gastric tumors to the small intestine by a form of metastasis other than peritoneal metastasis is rare.^10)^ The reason for the low incidence of metastatic small intestinal tumors, other than peritoneal metastasis, is thought to be that active peristalsis makes it difficult for cancer cells to implant.^26)^ Additionally, the small intestine is rich in immunoglobulin A and T lymphocytes, which contribute to its strong immune function.^27)^

In this case, we selected a combination of CPT-11 and capecitabine for chemotherapy. There have been several reports on chemotherapy and radiation therapy for carcinosarcoma; however, they are only case reports.^15,19)^ There have been only 6 cases wherein anticancer drugs were effective for the treatment of carcinosarcoma. A case was reported in which 5-fluorouracil (5-FU) was used to treat a postoperative recurrence of gastric carcinosarcoma differentiated into leiomyosarcoma, with the patient surviving for 31 months after recurrence.^28)^ Omori et al. reported a case in which a patient with liver metastasis from gastric carcinosarcoma that had differentiated into rhabdomyosarcoma survived for 7 months after receiving combined therapy with cyclophosphamide and docetaxel following intra-arterial infusion of epirubicin.^29)^ Selcukbiricik et al. reported a case in which a patient with gastric carcinosarcoma that differentiated into osteosarcoma survived for 14 months after receiving combined therapy with CDDP (cisplatin) and doxorubicin.^30)^ The efficacy of CPT-11 against gastric and gallbladder carcinosarcomas has also been reported.^16,31,32)^ There have also been reports of cases in which combination therapy with S-1 and CDDP, similar to that used for gastric cancer, was ineffective, but in which combination therapy with CPT-11 and Mitomycin C (MMC) was effective.^16)^ Based on these reports, we avoided the use of CDDP and prioritized the administration of 5-FU and CPT-11, introducing the XELIRI (capecitabine/irinotecan) regimen, a combination therapy of capecitabine and CPT-11, similar to that used for colorectal cancer.

This study has certain limitations. We speculated that intraluminal metastasis was present before the initial surgery. However, because small intestine imaging was not performed before the initial surgery, the possibility of tumors spilling out during surgery and subsequent implantation cannot be ruled out. As this is only a single case report and the prognostic information is also provisional, we cannot conclude from this case alone that XELIRI is an effective treatment for gastric carcinosarcoma. Owing to the possibility of future lymph node metastasis, caution must be exercised when concluding that local resection in this case as an appropriate treatment.

## CONCLUSIONS

We report a case of primary gastric carcinosarcoma with intraluminal metastasis to the small intestine. Because gastric carcinosarcoma often recurs via hematogenous metastasis, omitting lymph node dissection as palliative resection may be acceptable in the absence of definite lymph node metastasis. In addition, we experienced intraluminal metastasis to the small intestine, and depending on the case, it may be necessary to check for the presence or absence of bowel obstruction before surgery. In the present case, the patient presented with pulmonary metastasis, and XELIRI therapy was effective. However, the optimal chemotherapy regimen for the metastasis and recurrence of primary gastric carcinosarcoma remains unknown, and further investigation is required.
